# Augmented Renal Clearance Following Traumatic Injury in Critically Ill Patients Requiring Nutrition Therapy

**DOI:** 10.3390/nu13051681

**Published:** 2021-05-15

**Authors:** Roland N. Dickerson, Christin N. Crawford, Melissa K. Tsiu, Cara E. Bujanowski, Edward T. Van Matre, Joseph M. Swanson, Dina M. Filiberto, Gayle Minard

**Affiliations:** 1 Department of Clinical Pharmacy and Translational Science, College of Pharmacy, University of Tennessee Health Science Center, Memphis, TN 38163, USA; edward.vanmatre@uthsc.edu (E.T.V.M.); jswanson@uthsc.edu (J.M.S.); 2 Department of Pharmacy, JPS Health Network, Fort Worth, TX 76104, USA; CCrawfor04@jpshealth.org; 3 Department of Pharmacy, Methodist Charlton Hospital, Dallas, TX 75237, USA; meltsiu@gmail.com; 4 Department of Pharmacy, Huntsville Hospital, Huntsville, AL 35801, USA; cebujanowski@gmail.com; 5 Department of Surgery, College of Medicine, University of Tennessee Health Science Center, Memphis, TN 38163, USA; dfiliber@uthsc.edu (D.M.F.); gminard@uthsc.edu (G.M.)

**Keywords:** trauma, injury, kidney, creatinine, creatinine clearance, nutrition, augmented renal clearance, enteral nutrition, parenteral nutrition, protein

## Abstract

The intent of this study was to ascertain the prevalence of augmented renal clearance (ARC) in patients with traumatic injuries who require nutrition therapy and identify factors associated with ARC. Adult patients admitted to the trauma intensive care unit from January 2015 to September 2016 who received enteral or parenteral nutrition therapy and had a 24 h urine collection within 4 to 14 days after injury were retrospectively evaluated. Patients with a serum creatinine concentration > 1.5 mg/dL, required dialysis, or had an incomplete urine collection were excluded. ARC was defined as a measured creatinine clearance > 149 mL/min/1.73 m^2^. Two hundred and three patients were evaluated. One hundred and two (50%) exhibited ARC. A greater proportion of patients with ARC were male (86% vs. 67%; *p* = 0.004), had traumatic brain injury (33% vs. 9%; *p* = 0.001), a higher injury severity score (30 ± 11 vs. 26 ± 12; *p* = 0.015), were younger (36 ± 15 vs. 54 ± 17 years; *p* = 0.001), had a lower serum creatinine concentration (0.7 ± 2 vs. 0.9 ± 0.2 mg/dL; *p* = 0.001) and were more catabolic (nitrogen balance of −10.8 ± 13.0 vs. −6.2 ± 9.2 g/d; *p* = 0.004). The multivariate analysis revealed African American race and protein intake were also associated with ARC. Half of critically ill patients with traumatic injuries experience ARC. Patients with multiple risk factors for ARC should be closely evaluated for dosing of renally-eliminated electrolytes, nutrients, and medications.

## 1. Introduction

An erroneous assumption made by some clinicians is that patients with critical illness and without kidney dysfunction have normal glomerular filtration rates based on determination of a normal serum creatinine concentration. Recent evidence indicates that critically ill patients often have enhanced elimination of solutes, with a measured creatinine clearance (mCrCl) greater than anticipated when based solely on serum creatinine concentration or predictive formulas to estimate creatinine clearance. This physiologic process, via activation of renal functional reserve [1] by exogenous protein intake or mediators of the hypermetabolic-hypercatabolic response during critical illness, is referred to as augmented renal clearance (ARC) [2]. ARC has been reported in patients with traumatic injuries [3,4,5,6,7,8], burns [9,10], and sepsis [5,11,12,13]. The occurrence of this phenomenon during critical illness varies significantly, ranging from as low as 16% to as high as 100% of patients [11]. This wide variability may be explained by differences in the definition of ARC, patient populations, dosage of protein during nutrition therapy, and timing of the observation relative to intensive care unit (ICU) admission. The unanticipated presence of ARC could lead to the inability to achieve normal serum electrolyte concentrations with conventional electrolyte dosing [14,15,16,17,18,19,20], suboptimal dosing of beta-lactam antibiotic therapy during serious infections [12,21], or ineffective dosing for other renally-eliminated drugs or nutrients that cannot routinely be pharmacokinetically adjusted. The primary objective of this study was to ascertain the incidence of ARC for critically ill patients with severe traumatic injuries who require enteral or parenteral nutrition therapy. The secondary objective was to ascertain if any clinical features of patients with ARC differ from those without ARC.

## 2. Materials and Methods

Adult patients, at least 18 years of age, admitted to the Presley trauma intensive care unit (TICU) at Regional One Health in Memphis, TN, USA from January 2015 until September 2016, and who were referred to the Nutrition Support Service (NSS) for enteral or parenteral nutrition therapy were eligible for this retrospective study. Only patients who required enteral or parenteral nutrition therapy were included in this study as it is routine clinical practice of the NSS to collect a 24 h urine for determination of nitrogen balance and creatinine clearance and these patients exhibit high injury severity scores, are markedly catabolic, have a prolonged TICU duration of stay, and susceptible to infectious complications often requiring renally-eliminated antibiotic therapy [22]. Patients must have had a 24 h urine collection for determination of mCrCl and nitrogen balance (NBAL) within 4 days to 14 days after TICU admission. If more than one creatinine clearance measurement was conducted within the allotted time window, the first measurement was used for this analysis. Patients excluded were those with kidney injury as evidenced by a serum creatinine > 1.5 mg/dL or those who required hemodialysis or continuous renal replacement therapy prior to or during the timed urine collection, suspected rhabdomyolysis based on presence of urine myoglobin and an elevated serum creatine phosphokinase, a past medical history of chronic renal disease, or those who had an ad libitum oral intake.

Severe traumatic brain injury (TBI) was evident by a Glasgow Coma Scale (GCS) score of 3 to 8 prior to sedation or requirement for intracranial pressure monitoring. Patients were classified as septic by the presence of systemic inflammatory response syndrome and organ dysfunction due to an infection source. Pneumonia was confirmed via a diagnostic bronchoalveolar lavage with greater than 10^5^ colony forming units per mL in the effluent [23]. Injury severity score [24] (ISS) was obtained from the trauma registry at our institution. The sequential organ failure assessment (SOFA) [25] score was calculated at the time of the urine collection. This study was approved and conducted in accordance with the guidelines established by the university Investigational Review Board and hospital office of medical research. The requirement for informed consent was waived.

Measured variables were collected from the patient’s electronic medical record or NSS monitoring records. These variables included demographic information, nutrition markers, serum and urine chemistries, hematology and microbiology results, nursing records and clinical monitoring data, medications, past medical and surgical histories, and admission diagnoses. Serum electrolytes, creatinine, and urea nitrogen assays were conducted by the hospital laboratory using an automated analyzer as part of the patients’ routine clinical care. Patients had indwelling urinary catheters and the 24 h urine collection was obtained from midnight to midnight to coincide with the patient’s electronic fluid intake and output records. The urine collection was evaluated for completeness of the collection by members of the NSS at the time of the measurement. The urine collection was considered reliable if the laboratory volume and nursing record of urine output were within approximately 10% of each other, the mCrCl was at least 80% of predicted by the Cockcroft–Gault equation [26], the patient did not leave the TICU for a surgical procedure, and the patient’s nurse did not indicate any disposal of the urine collection [22]. Collections deemed incomplete or inaccurate by members of the NSS were not used for clinical purposes or in this analysis. The mCrCl was calculated using the following equation:

mCrCl (mL/min) = [uCr (mg/d)/(sCr(mg/dL) × 1440 min/d)] × 100 mL/dL(1)

where uCr is urinary creatinine excretion, sCr is serum creatinine, min is minutes, and d is day.

The CrCl calculation was then normalized to a body surface area of 1.73 m^2^ using the method of Mosteller [27]. 

Patients were preferentially given enteral nutrition by a naso or oro-enteric tube. Parenteral nutrition was employed if enteral nutrition was contraindicated or if the patient exhibited persistent gastric feeding intolerance despite prokinetic pharmacotherapy [28]. Caloric and protein goals were 30 to 32 kcal/kg/d and 2 to 2.5 g/kg/d, respectively [22]. Patients with obesity (body mass index > 29.9 kg/m^2^) were assigned calorie and protein goals of 22 to 25 kcal/kg ideal body weight (IBW)/d and 2 to 2.5 g/kg IBW/d of protein [29]. Pre-resuscitation body weight was used to determine target nutritional goals and estimation of creatinine clearance. Enteral or parenteral nutrition energy intake was reduced for patients who received a propofol infusion containing 10% lipid emulsion [30]. Blood glucose concentrations were maintained between 70 and 150 mg/dL by use of an intravenous regular human insulin infusion, intermediate acting insulin therapy, or sliding scale regular human insulin coverage [31,32,33]. The 24 h urine collection was used to determine NBAL and the protein in-take was adjusted accordingly to achieve nitrogen equilibrium (NBAL of −4 g/d to +4 g/d) if possible and without causing significant azotemia [22].

Data analysis was conducted using IBM SPSS Statistics version 27 (IBM Corporation, Chicago, IL) and SigmaPlot for Windows version 11.2 (Systat Software, Point Richmond, VA). The significance testing and reported probability values were two-sided for all variables. A *p* value of <0.05 was established as statistically significant. Any missing data were left blank and not used in the analysis. Continuous data were expressed as the mean ± standard deviation. Normality of the distribution of the data was evaluated by the Shapiro–Wilk test. Comparison of continuous variables between two independent groups was performed by the *t*-test or Mann–Whitney U test. The paired *t*-test or Wilcoxon signed rank test were used for comparing measured and predicted CrCL within the same set of patients. Categorical data were analyzed by Chi-square analysis. Goodness of fit of the linear models between mCrCl and continuous variables were evaluated using Pearson’s correlation analysis. Analysis of covariance was used to ensure any differences in mCrCl between those with and without ARC were not attributable to differences in predicted CrCl. A preliminary bivariate correlation analysis of all collected categorical and quantitative variables for the presence of ARC deficiency were conducted to determine which covariates to use in the binary logistic regression analysis. Those variables that achieved a statistically significant trend of *p* ≤ 0.10 from the univariate analysis, were used for the multivariate logistic regression analysis using a backwards stepwise elimination procedure to identify independent predictors of ARC. Independent predictors were expressed as the mean odds ratio (OR) with 95% confidence intervals. A receiver operating characteristic (ROC) curve was developed from the logistic multiple regression analysis model. Sensitivity, specificity, and positive and negative predictive values (PPV and NPV) were calculated for the mean, lower and upper 95% confidence intervals of the model from the ROC curve analysis.

## 3. Results

**Figure 1:**
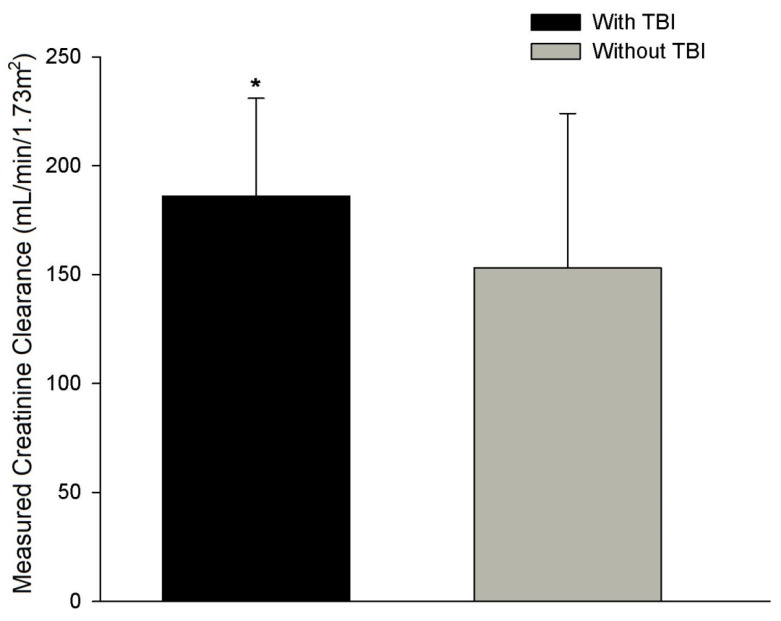
Measured creatinine clearance in patients with and without severe traumatic brain injury (TBI). * *p* = 0.004.

Two hundred and three patients were enrolled into this study. Most of the patients were male, ventilator dependent, admitted to the hospital due to injuries from a motor vehicle collision, received continuous enteral nutrition, and survived (Table 1). A mean ISS of 28 ± 12 for the entire population reflected patients with severe traumatic injuries. The 24 h urine collection was conducted 7 ± 3 days following admission to the TICU. One hundred and two out of 203 patients (50%) developed ARC with a mCrCl > 149 mL/min/1.73 m^2^ (125 or 62% of patients experienced a mCrCl > 129 mL/min/1.73 m^2^). A greater proportion of patients with ARC were male (*p* = 0.004), experienced severe TBI (*p* = 0.001), were younger (*p* = 0.001), were more tachycardic (*p* = 0.027), and had a higher ISS reflective of greater severity of injury (*p* = 0.015; Table 1). There was no difference for those with and without ARC in other markers of critical illness such as SOFA score, serum prealbumin and C-reactive protein concentrations, white blood cell count, minute ventilation, body temperature, or administration of corticosteroids, vasopressors, or neuromuscular blockers (Table 1). Despite similar calorie and protein intakes during the mCrCl determination, those with ARC were more catabolic as evidenced by a worsened NBAL (Table 2). Timing of the mCrCl determination between those with and without ARC were similar with mean observations conducted at ICU day 7 and 8, respectively (Table 2). There were no differences in clinical outcomes between groups except for the presence of pneumonia in those with ARC (Table 3). Those with severe TBI experienced nearly a four-fold increase in the presence of ARC (Table 1, *p* = 0.001) and had a significantly greater mCrCl when compared to those without severe TBI (Figure 1; *p* = 0.004). 

**Figure 2:**
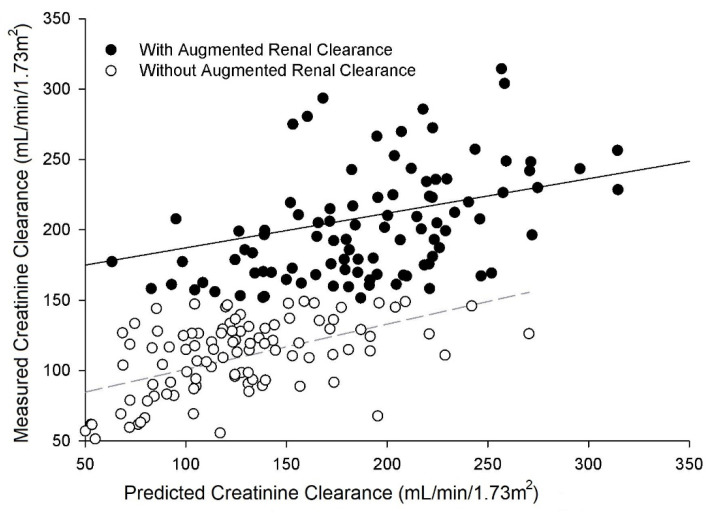
Relationship between measured (mCrCL) and predicted (Cockcroft–Gault equation) creatinine clearance (pCrCL) for patients with and without augmented renal clearance. Regression equations for each group, respectively, were: mCrCL ml/min/1.73 m^2^ = 0.25 × pCrCL (mL/min/1.73 m^2^) + 163 (*r* = 0.23, *p* = 0.018) and mCrCL mL/min/1.73 m^2^ = 0.32 × pCrCL (mL/min/1.73 m^2^) + 69 (*r* = 0.52, *p* = 0.001). The regressions for those with and without ARC, adjusted for predicted creatinine clearance, were significantly different from each other by ANCOVA (*p* = 0.001).

Serum creatinine concentration was significantly lower for those with ARC (*p* = 0.001) in addition to a lower serum urea nitrogen concentration (*p* = 0.001), and greater urine output (*p* = 0.002; Table 4). Mean mCrCl was significantly greater than predicted (pCrCL) by the Cockcroft–Gault equations for those with ARC (*p* = 0.029; Table 4). Measured CrCl increased as predicted CrCl increased for both those with and without ARC; however, the data indicated that the correlative relationship between mCrCl and pCrCl for both those with and without ARC was highly variable (*r* = 0.23 and 0.52, respectively; Figure 2). Those with ARC had a higher mCrCl across the range for predicted CrCl as evidenced by a higher y intercept by 94 mL/min/1.73 m^2^ in the regression equations resulting in a significant difference between groups (*p* = 0.001, Figure 2). 

**Figure 3:**
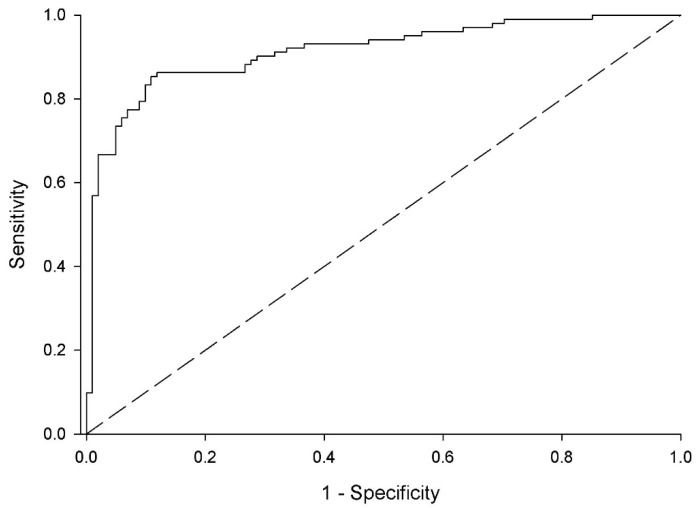
Receiver operating characteristic (ROC) curve of the logistic multiple regression analysis equation for determination of presence of ARC. Area under the ROC curve was 0.914 (95% confidence interval, 0.874, 0.955). *p* = 0.001.

Statistically significant variables associated with ARC, identified in the univariate correlation analysis, were given in Table 5 which included age, sex, serum creatinine and urea nitrogen concentration, presence of traumatic brain injury, pneumonia, and diabetes mellitus, urine output, body size, and ISS. In the stepwise multivariate analysis, significance of some of the variables in identifying those with ARC were sustained whereas new variables (with *p* values ranging from 0.05 to 0.10 in the univariate analysis) were added. Male sex, severe TBI, African American race, increased protein intake, worsened nitrogen balance, and younger age were associated with the presence of ARC (Table 6). Odds ratio and 95% confidence intervals for the significant variables in the multiple regression model are provided in Table 6. The ROC curve for the model is illustrated in Figure 3 and is depicted as follows: ROC score = (Sex (1 = male, 0 = female) × 2.151) − (age × 0.047) − (serum creatinine × 8.593) + (severe TBI (1 = present, 0 = absent) × 1.42) + (Protein intake g/kg/d × 0.722) − (NBAL g/d × 0.099) + (African American race (1 = present, 0 = absent) × 1.004) − (white blood cell count × 0.075) + 4.171.

Area under the ROC curve for the model was 0.91 (Figure 3; *p* = 0.001) indicating a strong association for the model for predicting the presence of ARC. Performance of the multivariate regression model for predicting the presence of ARC based on ROC values for the mean and asymptotic lower and upper bound 95% confidence intervals are given in Table 7.

## 4. Discussion

Our data indicate that ARC, as defined by a measured creatinine clearance > 149 mL/min/1.73 m^2^, is prevalent in approximately half of the patients admitted to the trauma ICU who exhibit marked protein catabolism (i.e., worsened nitrogen balance) requiring higher protein intakes, substantial ICU length of stay, and an extended duration of enteral or parenteral nutrition therapy. The Cockcroft–Gault equations, a common method for estimating creatinine clearance employed in clinical practice, significantly underestimated creatinine clearance for those with ARC. Significant factors associated with ARC include male sex, the presence of severe TBI, significant catabolism as evidenced by a negative nitrogen balance, increased protein intake, younger age, African American race, and a lower serum creatinine concentration. 

Some studies identify patients as having ARC when the mCrCl is ≥120 mL/min [4,11], 130 mL/min [2,5,6,7,8,12,13,34,35], 150 mL/min [3,21], or 170 mL/min [36] with divergence among studies whether to normalize the creatinine clearance to a body surface area of 1.73 m^2^. Normalization for body surface area, not consistently done in previous studies, is important due to the increasing prevalence of obesity in ICUs in the United States [29,37]. We used a threshold mCrCl of >149 mL/min/1.73 m^2^ for the definition of ARC as it has been shown to be more consistently associated with subtherapeutic serum concentrations from underdosing of renally-eliminated drugs when they are given at conventional doses [21,36,38,39,40,41].

ARC has occurred among various types of critically ill patients including those with trauma [4,5,7,8], TBI [3,42], thermal injury [9,39], hemorrhagic stroke [43], and sepsis [11,12,13,21,35,36,38,39,40,41]. Mechanisms for the development of ARC in critically ill patients is not entirely understood and likely due to multiple concurrent physiologic processes. One common pathophysiologic theme is the presence of a generalized inflammatory condition resulting in a hyperdynamic state with increased cardiac output, activation of renal functional reserve by increased renal blood flow and recruitment of residual nephrons beyond baseline, increased atrial natriuretic peptide, and increased production of cytokines and catecholamines [1,44]. Patients in our study consistently demonstrated evidence of a generalized inflammatory condition by an elevated c-reactive protein concentration, decreased serum prealbumin concentration, increased heart rate, and marked protein catabolism as evidenced by a negative nitrogen balance during nutrition therapy. 

The hyperdynamic, hypercatabolic flow phase following resuscitation is often prolonged in critically ill patients with multiple injuries due to their initial physical insult, subsequent multiple surgical procedures, and infectious complications [22]. Patients with a greater severity of injury (higher injury severity score) as well as the presence of severe TBI experienced a significantly higher mCrCl. Patients with ARC also exhibited a higher heart rate and increased urine output when compared to those without ARC. Finally, patients with ARC demonstrated more severe catabolism, based on a nitrogen balance determination, despite receiving a similar protein intake and comparable timing of the urine collection post admission to the ICU. Taken together, these data indicate that those with ARC were more hyperdynamic and hypercatabolic than those without ARC.

Another important etiology in the development of ARC, despite being overlooked in other studies examining ARC, is elicitation of renal functional reserve by increases in protein intake. Because of the marked catabolism following severe multiple traumatic injuries, an initial high target protein intake of 2 to 2.5 g/kg/d is recommended [22]. Evidence to support augmentation of glomerular filtration rate by increases in protein intake is derived from healthy subjects and hospitalized, non-critically ill patients who were given either oral diets or parenteral nutrition. In healthy subjects, creatinine clearance increased from 101 to 127 mL/min/1.73 m^2^ when the oral diet was advanced from 0.8 g/kg/d to 1.5 g/kg/d [45]. Increasing amino acid intake from 1 g/kg/d to 2 g/kg/d led to an increase in creatinine clearance from 102 to 143 mL/min in patients with Crohn’s disease during continuous parenteral nutrition [46]. Fliser and colleagues demonstrated a 16% increase in glomerular filtrate rate as measured by inulin clearance when healthy subjects were given an eight hour intravenous 1 g/kg amino acid infusion [47]. They also demonstrated an increase in renal plasma flow as determined by para aminohippurate clearance [47]. 

Although mean protein intakes during the urine collection were similar between those with and without ARC, increases in protein intake (g/kg/d) was a contributing factor towards identifying those with ARC in our multivariate model. To our knowledge, we are the first to demonstrate this association in critically ill patients with severe injuries and normal renal function who received enteral or parenteral nutrition support therapy. However, Doig and colleagues [48] demonstrated that a daily intravenous infusion of 100 g of amino acids significantly improved estimated glomerular filtration rate by approximately 8 mL/min/1.73 m^2^ when compared to standard care for critically ill patients with renal dysfunction.

As anticipated, those with ARC had significantly lower serum creatinine and urea nitrogen concentrations and were younger. Glomerular filtration rate declines with aging by approximately 1 mL/min/1.73 m^2^ per year after 40 years of age [49]. Following traumatic injuries, most older patients without evidence of chronic kidney disease or acute kidney injury, experience slightly higher, but clinically unappreciable, serum urea nitrogen concentrations than younger patients during nutrition therapy across a wide range of protein intakes [29,50]. These observations are likely due to the ability of healthy older patients to maintain renal functional reserve [47] in the absence of chronic renal disease and extra-renal diseases that may influence renal function. Male sex was also associated with a higher mCrCl presumably due to greater creatinine formation by more muscle mass for males versus females [49,51]. African American race has previously been determined to have an increase in estimated creatinine clearance by approximately 20% when derived from age, sex, and serum creatinine concentration [49] and also was a significant factor in our population for identifying those with ARC. 

The high incidence of ARC among critically ill patients with severe traumatic injuries in our study indicates marked potential for therapeutic underdosing of medications, electrolytes, and nutrients that are renally eliminated. The high prevalence of ARC among our patients with traumatic injuries may partially explain the requirement for aggressive intravenous electrolyte dosing in the management of hypophosphatemia [14,15,20], hypokalemia [18,52], hypocalcemia [16,17], and hypomagnesemia [19]. Subtherapeutic serum concentrations of renally-eliminated antibiotics, due to the presence of ARC in critically ill patients with infections, may lead to therapeutic failures, infection recurrence, and multidrug resistant strains [21]. 

A strength of this study includes the use of a 24 h urine collection that was evaluated for accuracy and completeness of the collection at the time of the measurement by members of the NSS. Accuracy was evaluated by comparison of nursing intake and output records with recorded laboratory volume, determination if the patient had left the unit for a procedure or surgery, and interview of the nurse caring for the patient for violations in collection procedures. A 24 h collection also ensures validity of the measurement as it avoids the potential for erroneous measurements with shorter collection times as the longer collection duration dampens the influence of normal diurnal variation in creatinine excretion [45] and reduces errors from short-lived activations in renal functional reserve due to intermittent enteral protein boluses commonly employed in clinical practice [22,29,30]. Our stricter definition of ARC (mCrCl > 149 mL/min/1.73 m^2^) along with an ample number of patients in each group provided greater clarity in identifying patients with substantial ARC where unintentional therapeutic underdosing of renally-eliminated medications, electrolytes, and nutrients may occur. This study has limitations. It was retrospective in design and limited to a single center, which may limit its generalizability to other trauma ICUs. Finally, lack of multiple measurements for each patient would have been useful to characterize the timing of initiation and duration of ARC during the patients’ ICU stay. 

## 5. Conclusions

Approximately half of critically ill patients with multiple traumatic injuries who receive enteral or parenteral nutrition experience ARC as defined as a measured creatinine clearance greater than 149 mL/min/1.73 m^2^. Proactive identification of those patients with ARC could serve to assist clinicians in effective dosing of renally-eliminated electrolytes, nutrients, and medications. Those patients with a combination of various risk factors for ARC such as younger age, high injury severity score, severe traumatic brain injury, significant protein intake, catabolism/negative nitrogen balance, pneumonia, male sex, African American race, tachycardia, increased urine output, and normal or low serum creatinine concentration should be closely evaluated for the potential presence of ARC. Our data provide further insight and facilitate heightened awareness to the clinician for identifying those critically ill patients with traumatic injuries who require parenteral or enteral nutrition therapy that may be experiencing ARC. 

## Figures and Tables

**Table 1:** Patient characteristics.

Variable	with ARC	without ARC	*p*
N	102	101	-
Male/Female, n/n	87/15	68/33	0.004
Race, AA/White/Other	36/57/9	34/60/7	0.209
Admitting diagnosis			
MVC	75	64	
GSW/KSW	14	14	
Assault/Fall	8	15	
Other	5	5	0.462
Age, years	36 ± 15	54 ± 17	0.001
Weight, kg	91 ± 22	97 ± 27	0.014
Height, cm	177 ± 9	174 ± 11	0.009
Body mass index, kg/m^2^	29 ± 16	32 ± 8	0.003
Body surface area, m^2^	2.10 ± 0.27	2.15 ± 0.31	0.083
Injury severity score	30 ± 11	26 ± 12	0.015
SOFA score	5.6 ± 1.8	5.9 ± 2.4	0.445
Ventilator dependent, *n* (%)	97 (95%)	95 (94%)	0.987
Severe traumatic brain injury, *n* (%)	34 (33%)	9 (9%)	0.001
History of diabetes mellitus, *n* (%)	8 (8%)	21 (21%)	0.015
Corticosteroids, *n* (%)	13 (13%)	13 (13%)	0.855
Vasopressors, *n* (%)	12 (12%)	21 (21%)	0.120
Neuromuscular blockers, *n* (%)	6 (6%)	11 (11%)	0.301
Beta adrenergic blockers, *n* (%)	53 (52%)	47 (47%)	0.941
Propofol, *n* (%)	40 (39%)	20 (20%)	0.004
Serum albumin, g/dL	2.6 ± 0.5	2.4 ± 0.4	0.001
Serum prealbumin, mg/dL	7.0 ± 3.6	6.7 ± 4.1	0.460
Serum C-reactive protein, mg/dL	21.3 ± 8.1	19.8 ± 9.4	0.259
Serum glucose *, mg/dL	126 ± 31	135 ± 35	0.026
White blood cell count *, cells/mm^3^	12.5 ± 4.9	12.8 ± 5.6	0.999
Serum 25-hydroxy vitamin D, ng/mL	19.9 ± 7.4	19.3 ± 6.5	0.593
Arterial pH *	7.42 ± 0.05	7.41 ± 0.06	0.105
Heart rate *, bpm	122 ± 29	115 ± 21	0.027
Minute ventilation *, L/minl	13.1 ± 5.0	13.3 ± 5.0	0.937
Maximum temperature *, °F	101.7 ± 1.3	101.3 ± 1.5	0.181

* on day of 24 h urine collection. AA, African American; ARC, augmented renal clearance as defined by a measured creatinine clearance > 149 mL/min/1.73 m^2^; bpm, beats per minute; GSW, gunshot wound, KSW, knife stab wound; L, liters; min, minute; MVC, motor vehicle collision; n, number; SOFA, sequential organ failure assessment. Variable missing data: injury severity score (*n* = 6), serum albumin (*n* = 21), serum prealbumin (*n* = 13), serum C-reactive protein (*n* = 14), serum 25-hydroxy vitamin D (*n* = 24), arterial pH (*n* = 16), and minute ventilation (*n* = 36).

**Table 2:** Nutrition therapy.

Variable	with ARC	without ARC	*p*
*N*	102	101	-
PN/EN/both	4/87/11	5/76/20	0.177
Nutrition therapy duration, d	16.5 ± 9.5	20.1 ± 13.2	0.027
Protein intake, g/d *	110 ± 62	98 ± 60	0.165
Protein intake g/kg/d *	1.4 ± 0.8	1.4 ± 0.8	0.122
Caloric intake, Kcals/d *	1237 ± 765	1088 ± 754	0.232
Caloric intake, Kcals/kg/d *	16 ± 10	15 ± 10	0.634
NBAL, g/d *	−10.8 ± 13.0	−6.2 ± 9.2	0.004
Hospital day of mCrCl, d	7 ± 3	8 ± 4	0.086

* on day of 24 h urine collection. ARC, augmented renal clearance as defined by a measured creatinine clearance > 149 mL/min/1.73 m^2^; d, days; EN, enteral nutrition; n, number; mCrCl, measured creatinine clearance; NBAL, nitrogen balance; PN, parenteral nutrition.

**Table 3:** Clinical outcomes.

Variable	with ARC	without ARC	*p*
*N*	102	101	-
Survived, *n* (%)	96 (94%)	89 (87%)	0.209
Sepsis, *n* (%)	56 (55%)	47 (46%)	0.293
Pneumonia, *n* (%)	54 (53%)	37 (36%)	0.028
Antibiotic days, d	9 ± 7	10 ± 9	0.330
Ventilator days, d	18 ± 18	20 ± 19	0.977
ICU length of stay, d	22 ± 16	24 ± 19	0.505
Hospital length of stay, d	35 ± 23	42 ± 38	0.217

ARC, augmented renal clearance as defined by a measured creatinine clearance > 149 mL/min/1.73 m^2^; d, days; ICU, intensive care unit; n, number.

**Table 4:** Renal function and measured creatinine clearance.

Variable	with ARC	without ARC	*p*
*N*	102	101	-
Hospital day of mCrCl, d	7 ± 3	8 ± 4	0.086
Serum creatinine, mg/dL	0.7 ± 0.2	0.9 ± 0.2	0.001
Serum urea nitrogen, mg/dL	16 ± 2	23 ± 12	0.001
Urine output, mL/d	3045 ± 1194	2305 ± 1195	0.001
Urine output, mL/kg/h	1.67 ± 0.69	1.36 ± 0.73	0.002
mCrCl, mL/min	255 ± 76	135 ± 37	0.001
mCrCl mL/min/1.73 m^2^	210 ± 38	109 ± 27	0.001
Predicted CrCl (Cockcroft–Gault), mL/min/1.73 m^2^	193 ± 55 *	125 ± 44 **	0.001

ARC, augmented renal clearance as defined by a measured creatinine clearance > 149 mL/min/1.73 m^2^; equation; CrCl, creatinine clearance; mCrCl, measured creatinine clearance. * *p* = 0.029 compared to mCrCl per 1.73 m^2^; ** *p* = 0.001 compared to mCrCl per 1.73 m^2^.

**Table 5:** Univariate correlation analysis between measured creatinine clearance and associated variables.

Variable	*r*	*p*
Age, years	−0.429	0.001
Sex, male	0.248	0.001
Weight, kg	−0.146	0.037
Height, cm	0.186	0.008
Body mass index, kg/m^2^	−0.247	0.001
Severe traumatic brain injury	−0.199	0.004
Diabetes mellitus	−0.142	0.043
Pneumonia	0.185	0.008
Injury severity score	0.142	0.047
APACHE II score	−0.186	0.008
Serum creatinine, mg/dL	−0.499	0.001
Serum urea nitrogen, mg/dL	−0.381	0.001
Urine output, mL/d	0.342	0.001
Urine output, mL/kg/h	0.247	0.001

APACHE, acute physiology and chronic health evaluation; *r*, Pearson’s correlation coefficient. Variable missing data: injury severity score (*n* = 6).

**Table 6:** Multivariate analysis of variables associated with augmented renal clearance. Data given as the mean (95% confidence interval).

Variable	Odds Ratio	*p*
Male sex	8.59 (2.69, 27.41)	0.001
Age, years	0.95 (0.93, 0.98)	0.001
African American race	2.73 (1.06, 7.01)	0.037
Severe traumatic brain injury	4.14 (1.33, 12.90)	0.014
Protein intake, g/kg/d	2.06 (1.09, 3.91)	0.027
Nitrogen balance, g/d	0.91 (0.86, 0.95)	0.001
Serum creatinine, mg/dL	0.00 (0.00, 0.00)	0.001
WBC, cells/mm^3^	0.928 (0.85, 1.01)	0.096

WBC, white blood cell count.

**Table 7:** Performance of the multivariate regression model for predicting presence of ARC.

Model Score	Sensitivity	Specificity	Positive Predictive Value	Negative Predictive Value
−0.77 (mean)	92%	68%	75%	90%
−1.15 (95% CI)	86%	87%	87%	86%
−0.06 (95% CI)	67%	97%	96%	75%

CI, confidence interval.

## Data Availability

The dataset used and analyzed for the current study is available from the corresponding author upon reasonable request.

## Implications

The results of this study provide insight into risk factors associated with augmented renal clearance in critically ill patients with traumatic injuries whereby increased doses of renally-eliminated electrolytes, medications, and nutrients may be necessary to obtain the desired therapeutic effect. Close monitoring of these patients is warranted.
